# Converting *Escherichia coli* isochorismatase YecD into γ-lactamase

**DOI:** 10.1186/s40643-025-00960-y

**Published:** 2025-10-25

**Authors:** Xiaoyan Guo, Yijie Tang, Xutao Zhao, Sheng Wu, Jianjun Wang

**Affiliations:** 1 College of New Materials and Chemical Engineering, Beijing Institute of Petrochemical Technology, Beijing, 102617 People’s Republic of China; 2 CAS Key Laboratory of Microbial Physiological and Metabolic Engineering, Institute of Microbiology, Chinese Academy of Sciences, Beijing, 100101 People’s Republic of China

**Keywords:** Active site constellation, ( +) γ-lactamase, Isochorismatase, Promiscuity

## Abstract

Five *Escherichia coli* proteins in the isochorismatase superfamily (EntB, RutB, Nic, YcaC, and YecD) were cloned and expressed. Among them, only RutB exhibited ( +) γ-lactamase activity. The primary structures of these five proteins were compared to those of a ( +) γ-lactamase (Mhpg) from *Microbacterium hydrocarbonoxydans*. Subsequently, the active site constellations (ASCs) of the proteins were superimposed. By imitating the ASCs of Mhpg and RutB, a single mutation converted YecD into an active ( +) γ-lactamase (YecD-G145C). A mutant with three mutations (YecD-G145C-W115E-V67I) engineered through combinatorial saturation mutagenesis was created. The catalytic efficiency (*k*_*cat*_*/K*_*m*_) of this mutant was 31-fold higher than that of YecD-G145C. Furthermore, the specific production rate (SPR) of the triple mutant (106 ± 4 mg/h·g dry cell weight, DCW) exceeded those of both RutB (89 ± 3 mg/h·g DCW) and Mhpg (46 ± 1 mg/h·g DCW), underscoring its superior catalytic robustness. The discovery of RutB and the latent ( +) γ-lactamase activity of YecD suggests that several members of the isochorismatase superfamily remain to be discovered, and members of this family could be used to identify novel ( +) γ-lactamases. Some of the members, such as YecD, could be engineered into robust catalysts.

**Graphical abstract:**

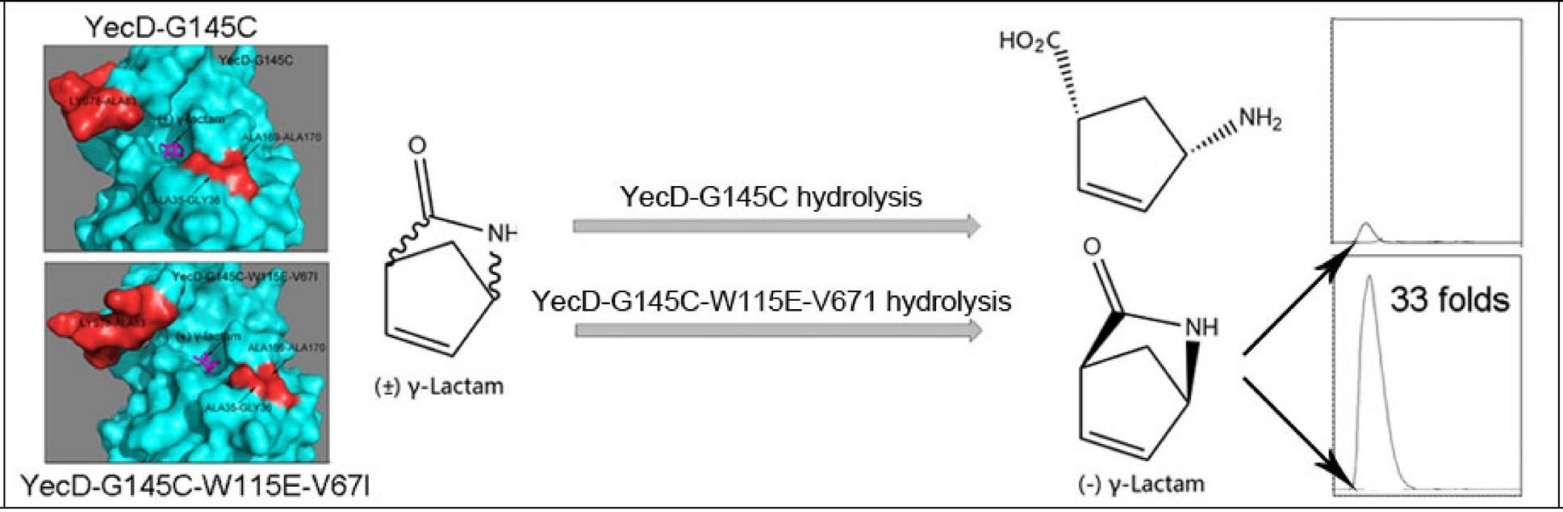

**Supplementary Information:**

The online version contains supplementary material available at 10.1186/s40643-025-00960-y.

## Introduction

γ-Lactamases (E.C.3.5.2.X) (Wang et al. 2010) are a group of enzymes used in catalyzing the preparation of optically pure 2-azabicyclo [2.2.1] hept-5-en-3-one (Vince lactam or γ-lactam). The chemical is an important chiral synthon for several high-value drugs (Singh & Vince 2012). Optically pure enantiomers of γ-lactam are prepared by chemical (King et al. 2000) and recrystallization methods (Tanaka et al. 2001). However, the enzymatic method (Taylor et al. 1993; Toogood et al. 2004) is the most preferred given its high efficiency, high productivity, and environmental friendliness.

Since the 1990s, γ-lactamases (( +) γ-lactamases and (−) γ-lactamases) have been discovered in various microbial strains (Gao et al. 2017; Kettle et al. 2015; Wang et al. 2015a, 2015b; Zhu et al. 2012). In our previous work, we identified Mhpg, a novel ( +) γ-lactamase, in *Microbacterium hydrocarbonoxydans* using the two-step method (Wang et al. 2015b). Mhpg was originally annotated as an isochorismatase in the GenBank. Later, RutB, a novel ( +) γ-lactamase and also an isochorismatase, was discovered in *E. coli* (Wang et al. 2015a). In a recent study by Gao et al. (Gao et al. 2017), another isochorismatase with ( +) γ-lactamase promiscuity was isolated from *M. hydrocarbonoxydans*implied. Enzyme promiscuity is thought to stem from the divergent evolution of enzymes from a common ancestor (Pandya et al. 2014). Consequently, it can be hypothesized that ( +) γ-lactamase activity was a promiscuous function of the progenitor of the isochorismatase superfamily.

The isochorismatase superfamily is a large group of enzymes. It is composed of five families, including nicotinamidase (E.C.3.5.1.19), nicotinamidase-related protein, N-carbamoylsarcosine amidohydrolase (E.C.3.5.1.59), isochorismatase (E.C. 3.3.2.1), and a family whose function remains unknown (Murzin et al. 1995). This superfamily is also annotated as the cysteine hydrolase superfamily because most of its members possess a conserved cysteine residue at the active site (Caruthers et al. 2005). Mhpg and RutB are categorized as N-carbamoylsarcosine amidohydrolases (Kim et al. 2010; Wang et al. 2015b). However, ( +) γ-lactamase and isochorismatase are two distinct groups of enzymes (Table S1, Supplementary Materials). It’s unclear whether members of the other four groups of the isochorismatase superfamily, including nicotinamidases and isochorismatases, are also ( +) γ-lactamases, or if they could be converted to ( +) γ-lactamases.

Enzymes are classified into families based on evolutionary processes, primarily through divergence from a common ancestor or convergence toward similar functions (Orengo & Thornton 2005), and are often categorized based on their functional similarities (Todd et al. 2001). The presence of promiscuity in one member of an enzyme family suggests that other members are likely to possess similar catalytic functions. Given the high commercial value of optically pure γ-lactam, there have been extensive and continuous attempts to discover novel γ-lactamases. To date, the majority of γ-lactamases have been identified through screening of environmental microbes (Wang et al. 2010). This strategy involves a series of complex procedures, including screening, protein purification, and gene manipulation. Nevertheless, the screening of a protein pool is a more cost-effective and energy-efficient approach.

In light of our prior findings and a report by Gao et al. (2017), we hypothesized that the five families in the isochorismatase superfamily could be a promising pool of novel ( +) γ-lactamases. In *Escherichia coli,* the representative proteins in these five families are Nic, YecD, RutB, EntB, and YcaC, respectively (Table S1, Supplementary Materials). To verify the above presumption, the five representative proteins were cloned and expressed in their soluble forms. Of these, only RutB exhibited ( +) γ-lactamase activity as previously reported (Wang et al. 2015a). Following a comparative analysis of the primary structures and active site configurations (ASCs) of the five proteins, YecD was selected for further study due to its ASC being the most similar to that of RutB. The protein was transformed into an active ( +) γ-lactamase by engineering it to imitate the ASCs of the two established ( +) γ-lactamases, Mhpg and RutB. Moreover, the catalytic efficiency of the YecD triple mutant (YecD-G145C-W115E-V67I) was significantly higher, approximately 31 times higher than the non-mutant form.

## Material and methods

### Chemicals, bacterial strains, plasmids, and culture media

Racemic Vince lactam was purchased from Acros (Beijing, China). All the other reagents were of analytical grade. For routine culture, *Escherichia coli* K-12 MG1655 (ATCC 47076) was cultivated at 37 °C in Luria–Bertani (LB) broth or on LB agar plates. YecD was expressed in *E. coli* TOP10 cells (TransGen Biotech, Beijing, China). The gene was first cloned into the pBAD/MCS plasmid (Invitrogen, CA, USA), which was then transformed into *E. coli* TOP10. Recombinant clones were selected on LB medium supplemented with 50 µg/ mL ampicillin.

### Extraction and purification of DNA

Genomic DNA of *E. coli* K-12 MG1655 was extracted using the TIANamp Bacterial DNA Kit (Tiangen, Beijing, China) following the manufacturer’s instructions. For plasmid recovery, cells were processed using the TIANprep Mini Kit, and recovery of DNA fragments was performed using the TIANgel Midi Kit. All the processes were performed according to the manufacturer’s instructions.

### Gene cloning and expression in *E. coli*

The genes *entB*, *rutB*, *nic*, *ycaC,* and *yecD* were amplified separately from the genomic DNA of *E. coli* str. K-12 substr. MG1655. The primers used along with the corresponding gene targets are listed in Table S2 (Supplemental Material). The size and genomic locations of the genes are also listed in Table S2. The PCR procedure was performed under the following conditions: initial denaturation at 95 °C for 5 min; 30 cycles of denaturation at 95 °C for 1 min, annealing at 53 °C for 1 min, and extension at 72 °C for 1.5 min. The final extension was performed at 72 °C for 10 min. The 2 × Phanta Flash Master Mix (Vazyme, Beijing, China) was used in the PCR process. The amplified fragments were individually cloned into the pBAD/MCS plasmid between the NdeI and HindIII restriction sites. The constructed plasmids were designated pBADnic, pBADyecD, pBADrutB, pBADentB, and pBADycaC. The recombinant plasmids were then introduced into *E. coli* TOP10 competent cells and cultured overnight at 37 °C in LB medium. A 5-mL aliquot of this pre-culture was inoculated into 500 mL of fresh LB broth and incubated until the cell density reached an OD580 nm of 0.8. The induction of protein expression was achieved by incorporating arabinose at a final concentration of 1.0 mM, followed by a 12-h extension of the incubation period. Subsequently, the cells were collected through centrifugation at 4000 × g for 10 min.

### Purification of His-tagged proteins on nickel (Ni)-chelating columns

The His-tagged protein was purified using Ni–NTA affinity chromatography as described previously (Guo et al. 2022). Briefly, 500 mL of culture was centrifuged, and the resultant cell pellets were resuspended in 40 mL of binding buffer (50 mM NaH_2_PO_4_, 300 mM NaCl, and 10 mM imidazole; pH 8.0). The cells were then lysed through ultrasonication on ice, and the resulting lysate was centrifuged at 10,000 × g to pellet the cell debris. The supernatant was then pre-equilibrated in a Novagen His∙Band gravity column. The column was washed with 20 mL of wash buffer (50 mM NaH_2_PO_4_, 300 mM NaCl, and 20 mM imidazole; pH 8.0), and the target protein was eluted using 10 mL of elution buffer (50 mM NaH_2_PO_4_, 300 mM NaCl, and 200 mM imidazole; pH 8.0). The eluate was desalted on a HiTrap desalting column (GE Healthcare), equilibrated with 50 mM Tris–HCl buffer (150 mM NaCl, pH 7.5), and dialyzed in 20 mM phosphate buffer (pH 7.2) for 24 h. The buffer was changed every 8 h.

### Building of the ASCs

The molecular structure and catalytic mechanism of isochorismatase OAIHL (PDB number 3LQY) have been described extensively (Goral et al. 2012). Therefore, OAIHL was used as the reference protein for building ASCs. The OAIHL catalytic triad comprises Asp14-Lys93-Cys126, and two residues, Gln16 and His59, which participate in substrate stabilization. Collectively, these five residues form a distinct spatial arrangement that constitutes the active site constellation (ASC). The equivalent of ASC residues in other enzymes was identified through sequence alignment (Table 1). The structure configuration, alignments, and RMSD calculations of the ASCs were performed using the PyMOL software (DeLano 2002).

**Table 1:** Catalytic and substrate-stabilizing residues in the active site constellations (ASCs)

Enzyme	Catalytic residues	Stabilizing residues
RutB*	Asp25-Lys134-Cys167	Gln27, Asn73
Mhpg*	Asp9-Lys84-Cys118	Gln11, His51
Nic*	Asp10-Lys111-Cys156	Gln12, Asp52
YecD	Asp25-Lys112-Gly145	Gln27, Val67
EntB	Asp37-Lys123-Gly156	Gln39, Gln79
YcaC	Asp19-Ala83-Cys118	Gln21, Ser59

^*^The data was sourced from (Guo et al. 2022)

### Homology modeling and molecular docking

The PDB files of EntB (2FQ1), YcaC (1YAC), and YecD (1J2R) were downloaded from the RSCB Protein Data Bank (Berman et al. 2000). Homology modeling of RutB was performed based on the SWISS-MODEL using 3IRV, with the cysteine hydrolase from *Pseudomonas syringae* pv. *phaseolicola* 1448A (PSPPH) as a template. The template used for Nic homology modeling was 2WTA, a nicotinamidase from *Acinetobacter baumanii*. Enantiomers of γ-lactam were docked into the catalytic site of YecD-G145C by an AutoDock plugin (Seeliger & de Groot 2010) of PyMOL software (DeLano 2002). As part of the docking process, the Solis and Wets local search method was used for conformation search, and the Lamarckian genetic algorithm (LGA) was used to investigate enzyme–substrate interactions (Huey et al. 2007). The enzyme–ligand interaction energies were calculated using an AutoDock-generated grid. The size of the grid was set to 126 × 126 × 126 points, and the grid space was set at 0.375 Å. The AutoDock method generated five enzyme–substrate configurations for ( +) γ-lactam docking. In these configurations, the nucleophilic attack distance between the thiol group of the Cys145 residue and the carbonyl carbon (C6) of ( +) γ-lactam was less than 3.5 Å. These configurations were putative rational poses. The global structure with the lowest energy was selected for subsequent analysis.

The volume of the ( +) γ-lactam molecule was measured using Discovery Studio 2019 software (BIOVIA Discovery Studio 2019, Dassault Systèmes, San Diego, CA, US).

### Construction of YecD-G145C mutant

The YecD-G145C variant was constructed by site-directed mutagenesis using the QuickChange method (Hogrefe et al. 2002), with plasmid pBADyecD as template. The primers used (P11–P12) are listed in Table S2. The 25 µL PCR mixture comprised 2.5 µL of 10 × KOD buffer, 1 µL of 25 mM MgCl_2_, 5 µL of a 2 mM dNTP mix, 0.5 µL of each primer (2.5 µM), 1 µL of template plasmid (10 ng/µL), and 1 U of KOD DNA polymerase. The thermal cycling protocol included an initial denaturation at 94 °C for 10 min, followed by two consecutive rounds of amplification. The first round involved 5 cycles of denaturation at 94 °C for 40 s, annealing at 53 °C for 40 s, and extension at 68 °C for 50 s. The second round of 25 cycles was performed under the following conditions: denaturation at 94 °C for 1 min, annealing at 60 °C for 1 min, and elongation at 68 °C for 6 min. A final extension was performed at 72 °C for 30 min. To eliminate the template plasmid, 10 µL of the PCR product was treated with 1 U of DpnI (New England Biolabs, Beijing, China) prior to transformation into *E. coli* TOP10 cells. Successful introduction of the mutation was verified by DNA sequencing of selected clones.

### Construction of combinatorial mutant libraries targeting positions 33/115 and 67/115 in YecD-G145C

Combinatorial saturation mutagenesis libraries were constructed through PCR-mediated randomization of target positions 33/115 and 67/115 using NNK-degenerate primer pairs (P15/P17 and P16/P17, respectively; Table S2). The pBADyecDG145C plasmid was the template DNA for PCR amplification. PCR was performed using the method described for gene cloning and expression in *E. coli*. Following amplification, the mutagenic fragments were purified using gel electrophoresis and assembled with the PCR-linearized pBADyecDG145C backbone (designed to replace the wild-type gene) using Gibson Assembly (Gibson et al. 2009). The assembled constructs were transformed into chemically competent *E. coli* TOP10 cells and plated for library generation. Individual clones were arrayed in 96-well plates containing 100 µL of LB medium supplemented with 50 µg/mL ampicillin. Cultures were grown at 37 °C for 18 h under constant 200 × g orbital shaking to reach the stationary phase. Glycerol (20 µL of 50% (v/v) was added to each well prior to cryopreservation at − 80 °C for long-term storage. Successful introduction of mutation incorporation was verified by sequencing of randomly selected clones, which also confirmed both the desired mutations and library diversity.

### High-throughput screening of mutant proteins

Screening for acyl transferase activity in the mutant libraries was performed using a high-throughput assay as previously described (Wang et al. 2015b). Briefly, 75 µL of a reaction mix composed of 25 µL hydroxylamine hydrochloride (1 M, pH 7.0, adjusted with 10 N NaOH), 25 µL sodium phosphate buffer (100 mM, pH 7.0), and 25 µL of either 20 mM S-lactamide or R-lactamide was added to 25 µL of cell suspensions from library clones grown in 96-well plates. The mixture was incubated at 30 °C for 2 h under constant shaking at 1000 rpm. Then, 200 µL of acidic FeCl_3_ solution (355 mM FeCl_3_ in 0.65 M HCl) was added to each well. The mixture was centrifuged at 3500 × g for 30 min to pellet cells, and the supernatant was transferred to a new microplate. Absorbance was measured at 500 nm using a BioTek Synergy 2 microplate reader (BioTek, Beijing, China). Controls (without resting cells) were included in each batch. Initial tests confirmed that YecD-G145C exhibited approximately four times higher activity toward S-lactamide than toward R-lactamide. A correlation was observed between the γ-lactamase and acyl transfer activities of RutB for S-lactamide, consistent with previous findings (Wang et al. 2015b). Based on these results, variants displaying both higher activity and improved enantioselectivity than YecD-G145C were selected for further evaluation in the kinetic resolution of racemic γ-lactam substrates. The enantiomeric composition of the resulting products was analyzed by chiral HPLC.

### MD simulations

The simulation of molecular dynamics (MD) was performed using GROMACS 2022 (Van Der Spoel et al. 2005). The protein–ligand complexes were solvated in a dodecahedral water box with TIP3P water molecules, and appropriate concentrations of Na⁺and Cl⁻counterions were used to neutralize the systems. Before production runs, each system underwent energy minimization (steepest descent algorithm, 5000 steps) to eliminate steric clashes, followed by a two-step equilibration: (1) 100 ps NVT ensemble equilibration using the Berendsen thermostat (300 K), and (2) 100 ps NPT ensemble equilibration employing the Parrinello-Rahman barostat (1 bar). Production MD simulations were carried out for 200 ns per system, with coordinates saved every 10 ps for subsequent analysis. Trajectory analyses were conducted using built-in GROMACS utilities, while binding pocket volume (BPV) calculations were performed with POVME 3.0 (Wagner et al. 2017). All graphical representations were generated using SigmaPlot 15.0 (Systat Software Inc., San Jose, CA).

### Molecular mechanics Poisson-Boltzmann surface area determination

The binding free energies of protein–ligand complexes were calculated using the Molecular Mechanics Poisson-Boltzmann Surface Area (MMPBSA) method (Kumari et al. 2014). For reliable estimation, a representative conformational snapshot exhibiting stable binding interactions was extracted from the equilibrated molecular dynamics trajectories. All energy calculations were performed using the g_mmpbsa toolkit (Kumari et al. 2014).

### Standard reaction conditions and γ-lactamase activity assay

The γ-lactamase activity was determined using a method previously described (Guo et al. 2022). Briefly, 40 μg of enzyme was added to 200 μL of a 4.6 mM racemic γ-lactam substrate solution (in 50 mM Tris–HCl, pH 8.0) and incubated at 35 °C for 5 min. After the reaction, the mixture was extracted with 100 μL of ethyl acetate and analyzed by HPLC (Waters, Massachusetts, USA) equipped with a CHIRALPAK AS-H column (250 × 4 mm; Daicel Chemical Industries, Tokyo, Japan). Separation was achieved using a mobile phase consisting of acetonitrile and isopropanol (90:10, v/v) at a flow rate of 1.0 mL/min, with detection performed at 230 nm. Enzyme activity (U) was defined as the amount of enzyme required to convert one nanomole of substrate per minute. Enantiomeric excess (*ee*) was calculated as *ee* = 100 × (A − B)/(A + B), where A and B represent the amounts of the two enantiomers.

### Determination of protein concentration and SDS-PAGE

The concentration of protein was determined using the bicinchoninic acid (BCA) assay kit (Thermo Fisher Scientific, Beijing, China). Bovine serum albumin (BSA) was used as the standard. Proteins were separated using SDS-PAGE using the Laemmli method, in which a 6% stacking gel and a 12% resolving gel were used (Laemmli 1970). The purity of the protein was assessed using Glyko BandScan software (Glyko, Novato, USA).

### Software and online service

The homology analyses of proteins were conducted using BLASTX (Altschul et al. 1990), while sequence alignments were performed using the Vector NTI 8.0 (InforMax, Inc.). Molecular graphics and analyses were visualized with PyMOL (DeLano 2002).

## Results

### Enzyme expression, purification, and determination of γ-lactamase activity

**Fig. 1:**
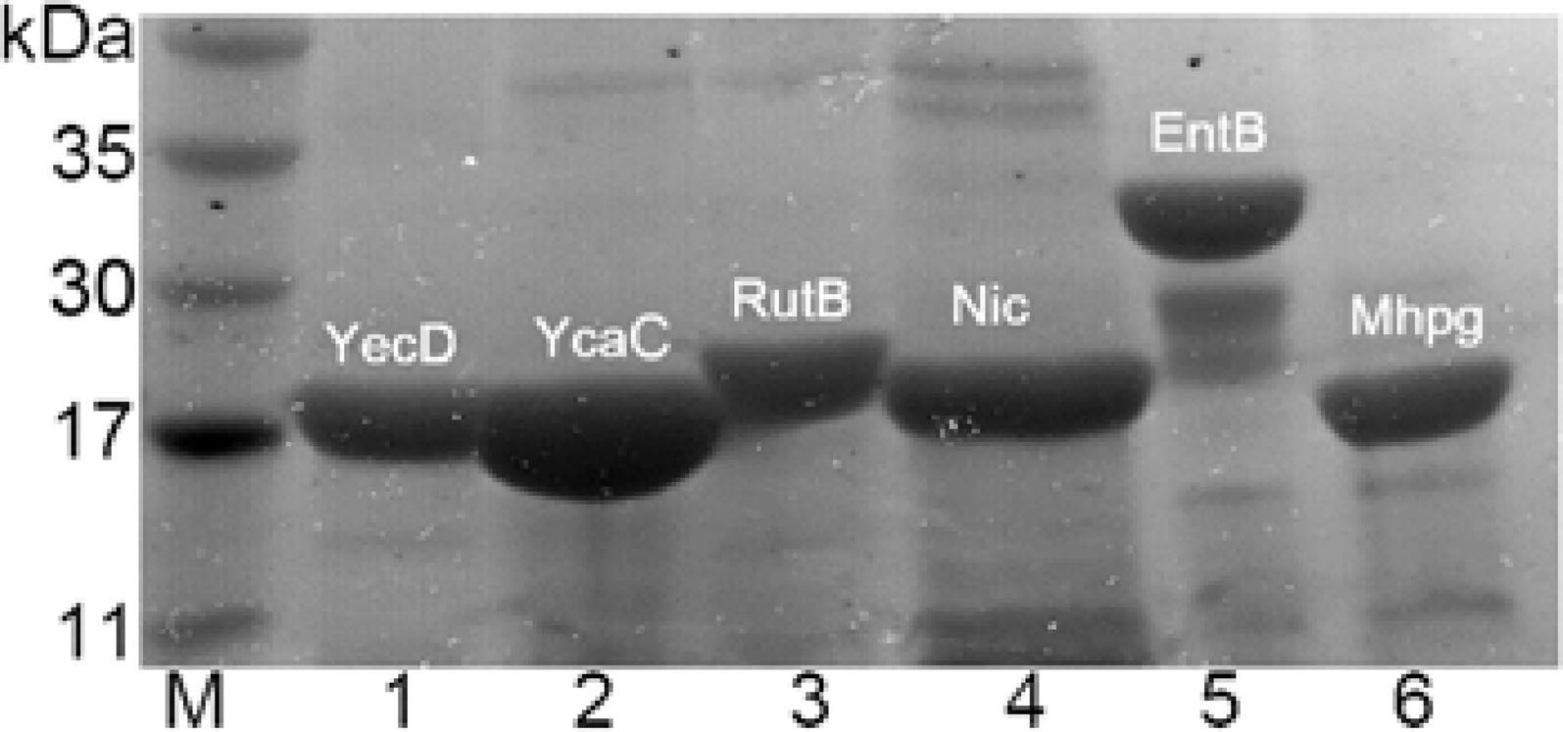
SDS-PAGE of purified proteins. M. SDS-PAGE Protein marker, 1. YecD (purity 91%), 2. YcaC (purity 85%), 3. RutB (purity 81%), 4. Nic (purity 73%), 5. EntB (purity 74%), 6. Mhpg (purity 78%). The purities of the proteins were estimated using the Glyko BandScan software. Around 20 µg of each protein was loaded, and the purity of the proteins was estimated using BandScan software

The isochorismatase superfamily of *E. coli* comprises five distinct subfamilies. The representative enzymes from each of the subfamilies include EntB (EC 3.3.2.1, isochorismatase), RutB (EC 3.5.1.59, N-carbamoylsarcosine amidohydrolase), Nic (EC 3.5.1.19, nicotinamidase), YcaC (uncharacterized), and YecD (nicotinamidase-related protein). The genes that code these proteins (*entB*, 861 bp; *rutB*, 696 bp; *nic*, 642 bp; *ycaC*, 627 bp; *yecD*, 603 bp) were cloned and expressed with a His tag in their C-termini. Most of the recombinant proteins were successfully expressed in the soluble fraction of *E. coli*. The proteins were purified using Nickel-affinity chromatography, and their molecular masses (EntB (~ 33 kDa), RutB (~ 26 kDa), Nic (~ 23 kDa), YcaC (~ 23 kDa), and YecD (~ 22 kDa) (Fig. 1)) determined using SDS-PAGE. Screening for ( +) γ-lactamase activity revealed that only RutB exhibited this catalytic function, consistent with our previous findings (Wang et al. 2015b).

### Identification of active site constellation by sequence alignment

The enzyme Mhpg from *Microbacterium hydrocarbonoxydans* harbors a putative catalytic triad (Asp9-Lys84-Cys118) along with two substrate-stabilizing residues (Gln11 and His51). Similarly, RutB from *E. coli* contains an analogous catalytic triad (Asp25-Lys134-Cys167) and two substrate-binding residues (Gln27 and Asn73). Together, these five residues form the active site constellation (ASC). When each of these residues in Mhpg and RutB was individually mutated to alanine, the ( +) γ-lactamase activity of the resulting variants was completely abolished (Wang et al. 2015a, 2015b), and this underscores their essential role in catalysis.

**Fig. 2:**
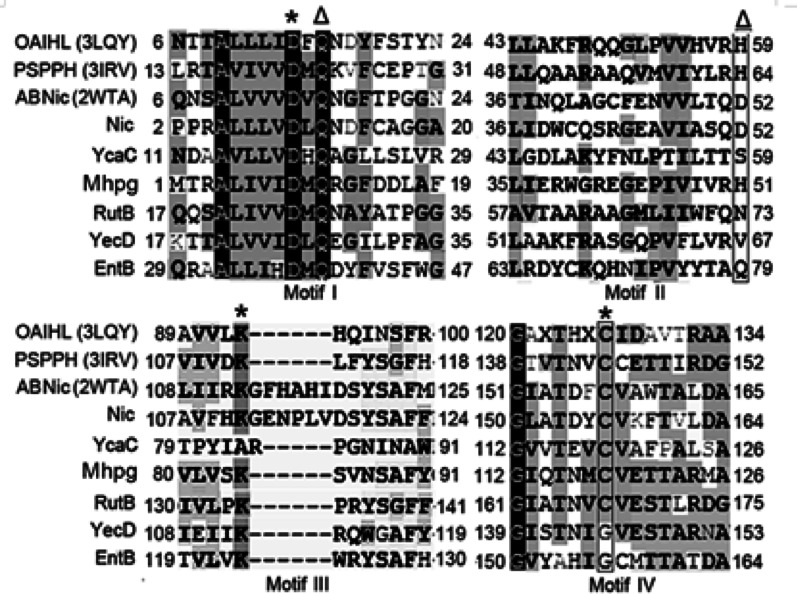
Multiple sequence alignments of proteins in the isochorismatase superfamily. The proteins contain four motifs. The asterisk represents residues constituting the catalytic triads in Motif I, Motif III, and Motif IV. The triangles represent two substrate-stabilizing residues in Motif I and Motif II. Abbreviations: OAIHL: isochorismatase from *Oleispira antarctica* (PDB number 3LQY); PSPPH: cysteine hydrolase from *Pseudomonas syringae* pv. *phaseolicola* 1448A (PDB number 3IRV); ABNic: isochorismatase from *Amycolatopsis benzoatilytica* (accession number WP_020657311); Nic: nicotinamidase from *E coli.* (accession number NP_416282); YcaC: *E*. *coli* YcaC (accession number AAC73983); Mhpg: amidase from *Microbacterium hydrocarbonoxydans* (accession number AIK97754.1); RutB: RutB from *E. coli* (accession number WP_001393558.1); YecD: isochorismatase from *E. coli* (accession number WP_022887063); EntB: isochorismatase from *E*. *coli* (accession number AAC73696)

Among the six proteins analyzed (Mhpg, EntB, RutB, Nic, YcaC, and YecD), YecD and EntB exhibited an Asp-Lys-Gly triad, whereas the others featured either an Asp-Lys-Cys triad (Mhpg, RutB, Nic) or an Asp-Ala-Cys triad (YcaC) (Table 1). Sequence alignment of these six proteins, along with three additional homologs—OAIHL (PDB: 3LQY), PSPPH (the structural template for RutB), and ABNic (the template for Nic) revealed four conserved motifs (Fig. 2). Among these, Motifs I, III, and IV were highly conserved and encompassed the catalytic triad residues (marked by asterisks in Fig. 2). In contrast, Motifs I and II contained the substrate-stabilizing residues (denoted by triangles in Fig. 2).

### Structure alignment of ASCs

**Fig. 3:**
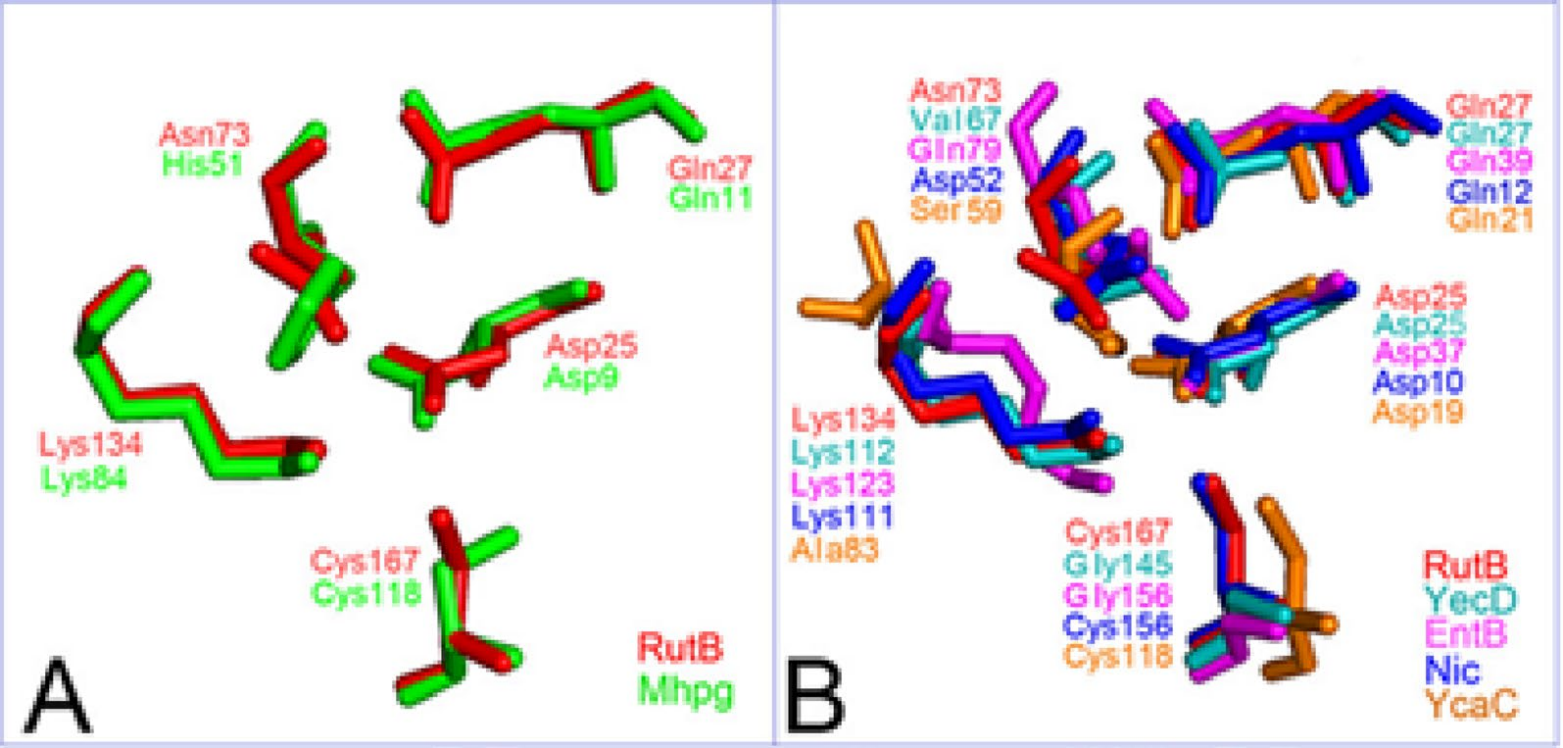
Superimposition of Active Site Constellations (ASCs). The PDB structure files of YecD (1J2R, cyan), EntB (2FQ1, deep purple), and YcaC (1YAC, orange) were directly downloaded from the RSCB Protein Data Bank. Homology modeling of Mhpg (green) was performed by SWISS-MODEL with 3MCW as template, which is a putative hydrolase of the isochorismatase superfamily from *Chromobacterium violaceum* ATCC 12472; the RutB (red) template was 3IRV, a cysteine hydrolase from *Pseudomonas syringae* pv. *phaseolicola* 1448A; the Nic (blue) template was 2WTA, which is a nicotinamidase from *Acinetobacter baumanii*. Structure alignment was performed using the PyMOL software

The spatial structures of the ASCs of Mhpg, RutB, YecD, EntB, and Nic were superimposed, and the RMSD values between the ASCs of enzymes were calculated (Table 2). Since the protein model of RutB was built using PSPPH (3IRV) as the template, the RMSD values between the ASC of PSPPH and other enzymes were calculated. The structures of ASCs for Mhpg, RutB, YecD, EntB, and Nic showed very high resemblance (Fig. 3). The constellation structures of the ASCs were most similar between RutB and YecD. The RMSD values between the ASC of 3IRV and those of RutB, and YecD were relatively low. However, the cysteine residue of the YecD and EntB triads was found to be replaced with glycine, while the lysine residue of the YcaC triad was found to be replaced with alanine (Fig. 3). The RMSD value (1.62 Å) between the ASCs of 3IRV and YcaC indicated that YcaC has the most unique ASC. As shown in Fig. 3, the Ala83 and Cys118 residues of YcaC were different from the conserved residue clusters. The ASC of Nic was similar to that of RutB and exhibited a relatively low RMSD value to 3IRV (Fig. 3B, Table 2). Despite this structural similarity between Nic and RutB, Nic is an inactive γ-lactamase.

**Table 2:** Root-mean-square deviation (RMSD) values of related proteins against the reference enzyme (3LQY)

Enzyme	RutB	Mhpg	Nic	YecD (1J2R)	EntB (2FQ1)		YcaC (1YAC)
RMSD to 3LQY (Å)	0.06	0.29	0.19	0.16	0.87	1.62

### Protein engineering of YecD-G145C and transformation

The above results revealed that the ASC of YecD is more similar to that of RutB than that of other members of the family. In other words, these two proteins are more likely to perform the same functions. A clear difference existed between ASCs of YecD and RutB. Specifically, the cysteine of the catalytic triad in YecD is substituted with glycine. Since cysteine is important for nucleophilic attack in substrate hydrolysis (Gao et al. 2017; Goral et al. 2012), the glycine residue was initially replaced with cysteine. This substitution introduced a detectable, albeit moderate, ( +) γ-lactamase activity in the YecD-G145C protein. No such activity was detected in the wild-type enzyme. Furthermore, the YecD-G145C mutant could hydrolyze several amide substrates that wild-type YecD would not (Table S3, Supplementary Materials).

**Fig. 4:**
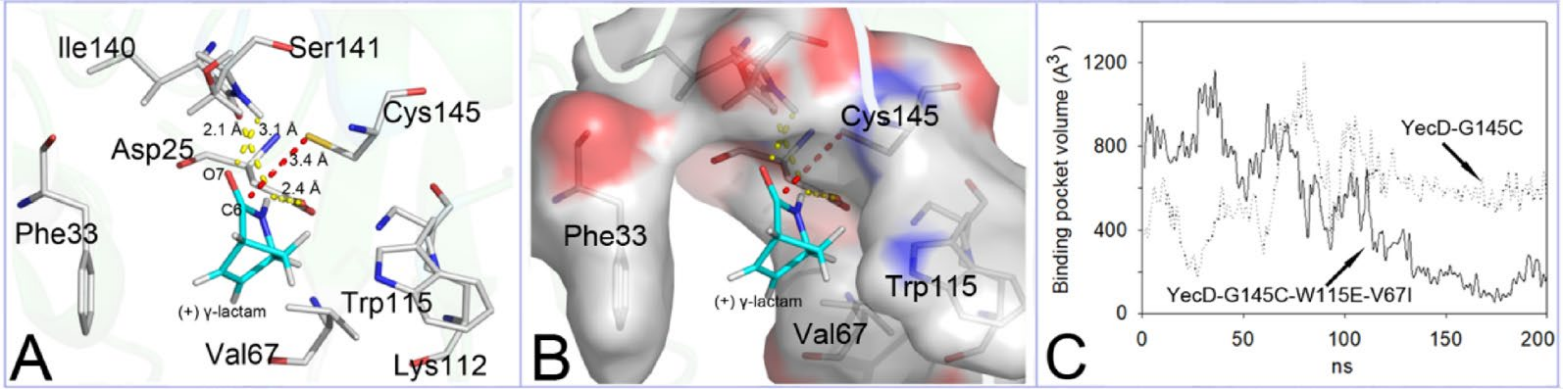
Docking ( +) γ-lactam into the binding pocket of YecD-G145C (**A**), the residues at the entrance of the binding pocket, and (**B**) the binding pocket volume (BPV) during MD. The yellow dashes represent hydrogen bonds, the red dashes represent the nucleophilic attack distance (between the sulfur atom of Cys145 and C6 of the ( +) γ-lactam) (**A**). Panels A and B are the same except that in panel B, a surface display mode is used to highlight the positions of related residues (**B**). The binding pocket volume (BPV) for YecD-G145C is represented by solid lines, while data for YecD-G145C-W115E-V67I are represented by dotted lines (C)

Although the YecD-G145C mutant exhibited ( +) γ-lactamase activity, its catalytic efficiency (*k*_cat_ = (1.4 ± 0.1) × 10^4^) was significantly lower than that of RutB (Wang et al. 2015a) (Table 3). To elucidate the structural basis for this difference, the two enantiomers of γ-lactam were docked into the binding site of the YecD-G145C mutant, and their binding modes were analyzed. As shown in Fig. 4A, the ( +) γ-lactam enantiomer assumed a productive binding pose within the active site. In this conformation, the thiol group of Cys145 was positioned 3.4 Å from the carbonyl carbon (C6) of the substrate, consistent with an optimal distance for nucleophilic attack (Gao et al. 2017; Goral et al. 2012). The carbonyl oxygen (O7) was stabilized by hydrogen bonds with the backbone amides of Ser141 and Cys145, forming a characteristic ‘oxyanion hole’ (Line et al. 2004). Further stabilization was provided by two additional hydrogen bonds between the substrate’s N–H hydrogen and the backbone carbonyls of Ile140 and Asp25 (Fig. 4A). In contrast, docking of the ( −) γ-lactam enantiomer did not yield a catalytically competent pose, explaining the experimentally observed stereoselectivity of YecD-G145C for the ( +) enantiomer.

**Table 3:** Kinetic parameters of YecD mutants in ( +) γ-lactam hydrolysis

Mutant	*K*_m_ [mM]	*k*_cat_ [min^−1^]	*k*_cat_ /* K*_m_ [M^−1^‧min^−1^]
YecD-G145C	72 ± 4	103 ± 5	(1.4 ± 0.1) × 10^3^
YecD-G145C- W115F	74 ± 3	317 ± 15	(4.3 ± 0.3) × 10^3^
YecD-G145C-W115Y	36 ± 3	393 ± 9	(10.9 ± 1.0) × 10^3^
YecD-G145C-W115E-V67I	16 ± 2	701 ± 30	(44 ± 6) × 10^3^
YecD-G145C- F33A	67 ± 4	98 ± 6	(1.5 ± 0.1) × 10^3^
YecD-G145C- V67A	70 ± 3	112 ± 13	(1.6 ± 0.2) × 10^3^
YecD-G145C- W115A	79 ± 3	107 ± 11	(1.4 ± 0.2) × 10^3^

The errors are the standard deviations of three independent experiments

Three residues (Phe33, Val67, and Trp115) formed a steric gate at the substrate entrance tunnel (Fig. 4B), with each residue being characterized by aromatic or branched side chains. The low catalytic efficiency of the mutant was initially hypothesized to be caused by obstruction of substrate diffusion by these amino acids. To validate this hypothesis, combinatorial saturation mutagenesis targeting these positions was performed. Library screening revealed that while the majority of variants (88.60%) showed diminished enzymatic activity and 11.32% maintained activity levels comparable to YecD-G145C, a small but significant fraction (0.05%, representing five out of 6,000 screened clones) exhibited enhanced catalytic efficiency (Table 3). Notably, Phe33 substitutions were absent among the improved variants, strongly suggesting that mutation at this position is either neutral or does not enhance the enzyme’s ( +) γ-lactam hydrolysis activity. This observation was further corroborated by kinetic analyses, which demonstrated nearly identical catalytic parameters between YecD-G145C and its F33A mutant. Further analysis of the active mutants (YecD-G145C-W115F, YecD-G145C-W115Y, and YecD-G145C-W115E-V67I) revealed that catalytic enhancement was achieved despite the introduction of alternative aromatic residues (in the case of W115F/Y) or branched side chains (in W115E-V67I). This observation, when combined with activity data obtained from alanine-substituted double mutants, led to the conclusion that the initial steric hindrance hypothesis might be incomplete.

The triple mutant YecD-G145C-W115E-V67I showed significantly higher activity toward specific amide substrates compared to the single mutant (Table S3). Both mutants showed similar activity toward small amides like dimethyl formamide and acetamide. The triple mutant exhibited significantly higher activity than the single mutant toward substrates like S-lactamide, achieving a 42.5% increase in catalytic efficiency. This substrate-dependent enhancement suggests that the W115E and V67I mutations not only optimize the binding pocket geometry for γ-lactam hydrolysis but also broaden the enzyme’s promiscuity toward structurally diverse amide compounds.

For large-scale biotransformation, whole cells of the YecD-G145C-W115E-V67I triple mutant were employed. A cell suspension containing 5 g (dry weight) of cells harvested from a 5-L batch culture was incubated with 100 ml of 2 M substrate solution (200 mM phosphate buffer, pH 7.0) at 35 °C for 16 h. After the reaction, the remaining substrate was recovered following established protocols (Wang et al. 2015a). The triple mutant demonstrated exceptional catalytic performance, yielding 8.5 g of product with 79% conversion efficiency and maintaining excellent stereoselectivity (ee > 99.9% for the (−)-γ-lactam enantiomer). The specific production rate reached 106 ± 4 mg/h·g, significantly outperforming the YecD-G145C, RutB, and Mhpg whole-cell catalysts in both productivity and yield (Table 4). Notably, all four proteins exhibited complete enantioselectivity, with no detectable ( +)-γ-lactam formation (Fig. S1, Supplementary Materials). These results demonstrate that the YecD-G145C-W115E-V67I triple mutant is an efficient and robust biocatalyst for (−)-γ-lactam production under mild conditions (Wang et al. 2015a).

**Table 4:** Comparative performance of enzymes in large-scale ( −)-γ-lactam production

Enzyme	Recovered product [g]	Yield [%]	*ee* value [%]	SPR [mg/h•g]
RutB	7.1 ± 0.3	65 ± 3	99.9	89 ± 3
Mhpg	3.7 ± 0.2	34 ± 2	99.9	46 ± 1
YecD-G145C	1.4 ± 0.1	13.2 ± 0.7	99.9	18 ± 1
YecD-G145C-W115E-V67I	8.6 ± 0.5	79 ± 5	99.9	106 ± 4

SPR: Specific production rate; *ee* value (enantiomeric excess value) for ( −) γ-lactam

### Unraveling the catalytic enhancement mechanism of the YecD-G145C/W115E/V67I mutant

Given that residue substitutions at the substrate entrance tunnel may alter the BPV, which in turn affects the catalytic efficiency of the mutant (Guo et al. 2022), the BPVs of the enzymes during MD simulation were analyzed using the POVME software. Subsequent to the initial dramatic fluctuations, the BPV of the YecD-G145C-W115E-V67I protein (the triple mutant) was sustained at a relatively stable level of approximately 200 Å^3^. A similar trend was observed for the YecD-G145C protein (the single mutant), which ultimately attained a substantially larger volume of 600 Å^3^ (Fig. 4C). Analysis with the Discovery Studio 2019 software revealed that the molecular volume of ( +) γ-lactam in the catalysis was approximately 98.8 Å^3^. This phenomenon was similar to that observed in Nic-del (Guo et al. 2022), which was found to possess a substantially more compact binding pocket in comparison to its wild-type counterpart. Consequently, a conducive environment for small substrate catalysis was established.

The binding free energy (ΔG), a key thermodynamic parameter for assessing protein–ligand interaction strength, was quantitatively determined using the gmx_mmpbsa package (Table S4, Supplementary Materials). Notably, the triple mutant (YecD-G145C-W115E-V67I) exhibited substantially stronger binding affinity for the ( +) γ-lactam substrate, with ΔG decreasing from − 4.4 kJ/mol in the single mutant to − 18.8 kJ/mol. This enhancement was primarily driven by a 15.4 kJ/mol reduction in the entropic penalty (− TΔS), and further supported by synergistic improvements in polar (PB) and non-polar (SA) solvation energies, as well as van der Waals (VDW) interactions (Table S4). These findings strongly suggest that the W115E and V67I mutations confer enhanced binding affinity through two distinct yet cooperative mechanisms: (1) rigidification of the binding interface to reduce entropic costs, and (2) optimization of local electrostatic and hydrophobic interactions (Chang et al. 2007; Genheden & Ryde 2015; Kumari et al. 2014).

## Discussion

### Screening of novel ( +) γ-Lactamases

The primary structure-sequence guided method was used to identify a novel ( +) γ-lactamase from *Bradyrhizobium japonicum* USDA 6 using the sequence of a ( +) γ-lactamase from *Sulfolobus solfataricus* as the template (Zhu et al. 2012). This method is based on the evolutionary relationships between proteins. However, our experimental results indicate that even when proteins share 60% sequence identity, the probability of obtaining inactive enzymes that are structurally identical to active enzymes is high (data not shown). Even though alternative approaches using tertiary structure alignment have been used to identify enzyme promiscuity (Sun et al. 2015), the limitation of this method is that an enzyme’s catalytic function depends primarily on the spatial arrangement of its ASC rather than the overall protein structure (Glasner et al. 2006). To address these challenges, a straightforward and effective screening strategy that combines primary sequence analysis with ASC comparison was developed. This approach specifically targets the catalytic residue architecture to identify functional ( +) γ-lactamases and reactivate inactive enzyme scaffolds through rational active site engineering. By focusing specifically on the catalytic residue arrangements rather than the whole sequence or structural features, functional ( +) γ-lactamases can be discovered more reliably through this method.

### Substrate entrance engineering is highly effective in enhancing the promiscuity of YecD-G145C

Three principal strategies, including (1) active site engineering (this study), (2) allosteric regulation (Süel et al. 2003), and (3) substrate entrance tunnel engineering (Gao et al. 2014; Mizuno et al. 2008), enhance enzyme promiscuity. The findings of the present study revealed that substrate entrance tunnel engineering effectively enhances enzyme promiscuity. Targeted mutagenesis of key entrance rim residues (Val67 and Trp115; Fig. 4) significantly enhanced the catalytic activity of YecD-G145C, demonstrating the potential of this engineering strategy. Interestingly, these improvements were observed despite the introduction of either aromatic (in double mutants) or branched (in triple mutants) amino acid mutations at the tunnel periphery. This is interesting because these modifications would normally be expected to create steric hindrance. Rather than impeding function, these residue changes promoted the formation of a more compact and complementary binding pocket through subtle architectural remodeling of the entrance tunnel. This optimized geometry reduces the substrate binding free energy (ΔG) through two key mechanisms: (1) improved shape complementarity between pocket and substrate, which reduces the entropic penalty of binding; and (2) enhancing molecular interactions through optimal contact distances. The resulting catalytic improvement likely arises from both superior substrate positioning and more thermodynamically favorable binding events, as confirmed by the substantial increase in catalytic efficiency.

While the structure-independent screening method applied in the present study provides an efficient way to convert inactive enzymes into functional ( +) γ-lactamases, structural studies remain essential for understanding the underlying catalytic mechanisms. Comparative analysis of RutB and the YecD mutant structures could explain why Nic remains inactive and why active site mutation only enhanced the enzymatic activity of YecD (but not EntB, data not shown). Additionally, structural characterization of the YecD triple mutant, currently underway in our laboratory through protein crystallization efforts, will help elucidate how the introduced mutations enhance catalytic activity by potentially revealing key conformational changes in the substrate entrance tunnel and the architecture of the active site. This combined approach of computational screening and structural validation offers a powerful strategy for enzyme discovery and engineering.

## Conclusions

The isochorismatase superfamily is a promising source of novel ( +) γ-lactamases. The methodology developed in this study, which combines primary sequence analysis with ASC comparison, can be effectively applied to other members of the isochorismatase superfamily to identify other ( +) γ-lactamases. Moreover, this approach has broader applicability and can be extended to other enzyme superfamilies to identify unknown catalytic promiscuity. The method is particularly valuable for revealing latent enzymatic activities, as demonstrated by the successful identification and activation of naturally inactive proteins like YecD. Some of these newly discovered enzymes may exhibit superior biocatalytic properties, including higher catalytic efficiency, improved stability, or broader substrate specificity.

## Supplementary Information

Supplementary Material 1 (DOCX 104 kb)

## Data Availability

The datasets generated and the materials are available from the corresponding author on reasonable request.
