# Supplementary material for: Converting Escherichia coli isochorismatase YecD into γ-lactamase
Source: Bioresour Bioprocess. 2025 Oct 25;12(1):118. doi: 10.1186/s40643-025-00960-y (PMC12553813; doi:10.1186/s40643-025-00960-y)
Supplement: Supplementary file 1 — Supplementary Material 1 (DOCX 104 kb) [file 40643_2025_960_MOESM1_ESM.docx]

**Supplementary Materials**

**Converting *Escherichia coli* isochorismatase YecD into γ-lactamase**

Xiaoyan Guo^1^, Yijie Tang^1^, Xutao Zhao^1^, Sheng Wu^2^, Jianjun Wang^2†^

^1^ College of Chemical Engineering, Beijing Institute of Petrochemical Technology, Beijing, 102617, P.R. China

^2^ CAS Key Laboratory of Microbial Physiological and Metabolic Engineering, Institute of Microbiology, Chinese Academy of Sciences,, Beijing, 100101, P.R. China

**Running title**: Converting *E. coli* YecD into γ-lactamases

^†^**Correspondence**: Jainjun Wang, CAS Key Laboratory of Microbial Physiological and Metabolic Engineering, Institute of Microbiology, Chinese Academy of Sciences,

Tel. 86-10-64807417. E-mail: [wangjj@im.ac.cn](mailto:wangjj@im.ac.cn)

**Assay for amidase activity**

The amidase activity was assessed using an adapted protocol based on Muftic's established procedure (Muftic, 1964). Each 100 μL reaction mixture, composed of 0.1 M potassium phosphate buffer (pH 8.0) and 40 μg of purified enzyme, was initiated by adding amide substrates to a final concentration of 10 mM and incubated at 30°C. At specific intervals, 20 μL samples were extracted and combined with 1 mL of sodium phenoxide/acetone reagent (comprising 675 mM NaOH, 64 mM phenol, and 136 mM acetone). To develop color, 1 mL of sodium nitroprusside (0.01% in acetate/HCl buffer, pH 6.0) and 1 mL of sodium hypochlorite (0.5%) were added sequentially. After 10 minutes of incubation at 30°C, the amount of ammonium ions generated was determined by measuring the absorbance at 625 nm and comparing to a standard curve prepared with (NH_4_)_2_SO_4_.

Table S1 Comparisons between γ-lactamase family and isochorismatase superfamily

| Superfamily | Family |  | E.C number | Catalyzed reaction | Representative protein (accession number) |
| --- | --- | --- | --- | --- | --- |
| - | γ-lactamase |  | E.C.3.5.2.X | γ-lactam hydrolysis | Mhpg (YP_004225617)  RutB (AAC74096) |
| Isochorismatase | Nicotinamidase |  | E.C.3.5.1.1 | Nicotinamide hydrolysis | Nic (NP_416282) |
|  | Nicotinamidase related protein |  | - | - | YecD (AAC74937) |
|  | N-carbamoylsarcosine amidohydrolase |  | E.C.3.5.1.59 | Carbamoylsarcosine hydrolysis | RutB (AAC74096) |
|  | Isochorismatase |  | E.C.3.3.2.1 | Isochorismatase hydrolysis | EntB (AAC73696) |
|  | unknown function family |  | - | - | YcaC (AAC73983) |

Table S2 Oligonucleotides for PCR amplification and genome location and gene length of genes in this study

| Name | DNA-sequence (5’-3’) | Description | Genome location  (bp number) | Gene length (bp) |
| --- | --- | --- | --- | --- |
| P1 | GGGAATTCCATATGGCTATTCCAAAATTACAGG | *entB* upstream | 627694- 628551 | 857 |
| P2 | CCCAAGCTTTTTCACCTCGCGGGAGAGTAGCTT | *entB* downstream |  |  |
| P3 | GGGAATTCCATATGACAACCTTAAC | *rutB* upstream | 1072171-1072863 | 692 |
| P4 | CCCAAGCTTAGCGATACGAGCAAA | *rutB* downstream |  |  |
| P5 | GGGAATTCCATATGCCCCCTCGCGCCCTGTTA | *nic* upstream | 1851887- 1852528 | 641 |
| P6 | CCCAAGCTTCCCCTGTGTCTCTTCCCAGTCTG | *nic* downstream |  |  |
| P7 | GGGAATTCCATATGACCAAACCGTATGTTCGTCTT | *ycaC* upstream | 944931- 945557 | 626 |
| P8 | CCCAAGCTTTTTCTGCTTCGTTAACGTGTCATA | *ycaC* downstream |  |  |
| P9 | GGGAATTCCATATGAGGCACGATTTTGTTTTTT | *yecD* upstream | 1950832-1951398 | 566 |
| P10 | CCCAAGCTTCGCGTTGAGGATCTCTTCCACGCTA | *yecD* downstream |  |  |
| P11 | TCTCGACCAATATCTGTGTTGAATCCACCGCC | *yecDG145C* upstream | - | - |
| P12 | GGCGGTGGATTCAACACAGATATTGGTCGAGA | *yecDG145C* downstream |  |  |
| P13 | ggtgttccataaaagttacagtgccttttt | *Nic-del* upstream |  |  |
| P14 | aaaaaggcactgtaacttttatggaacacc | *Nic-del* downstream |  |  |
| P15 | GAAGGCatcttacctnnkgccggtggtccacata | *yecD34NNK* upstream | - | - |
| P16 | GTGTttctggtgcgcNNKggctggtctgcc | *yecD67NNK* upstream |  |  |
| P17 | GTACCGTAAAACGCACCMNNTTGACGTTTGA | *yecD115NNK* downstream |  |  |

Table S3 Activity assays on amide substrates

| Substrate | YecD  Specific activity ( U/mg ) | YecD-G145C  Specific activity ( U/mg ) | YecD-G145C-W115E-V67I  Specific activity ( U/mg ) |
| --- | --- | --- | --- |
| Dimethyl formamide | ND | 8.0 ± 0.5 | 9.0 ± 0.3 |
| Urea | ND | ND | ND |
| Acetamide | ND | 12 ± 1 | 11 ± 1 |
| Propionamide | ND | 47 ± 3 | 50 ± 2 |
| Glutamine | ND | ND | ND |
| S-lactamide | ND | 40 ± 1 | 57 ± 1 |
| R-lactamide | ND | 10 ± 1 | 11 ± 0.3 |
| Valerolactam | ND | ND | ND |
| Caprolactam | ND | ND | ND |

ND, not detected.

Table S4 Calculation of binding energy using MMPBSA (energy unit: kJ/mol)

| Complex | dG | MM | TdS | PB | SA |
| --- | --- | --- | --- | --- | --- |
| COU + VDW | | | | | |
| YecD-G145C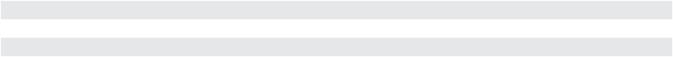 | −4.4 | −58.1  −11.5+ −46.7 | 28.8 | 36.3 | −11.4 |
| YecD-G145C-W115E-V67I | −18.8 | −53.0 | 13.4 | 32.6 | −11.8 |
| −8.8 + −44.2 | | | | | |

Abbreviations: COU, electrostatic energy; dG, protein-ligand-binding energy; MM, molecular mechanics vacuum energy; PB, polar solvation energy; SA, non-polar solvation energy; VDW, Van der Waals’ energy. Calculation of binding energy using MMPBSA (energy unit: kJ/mol).


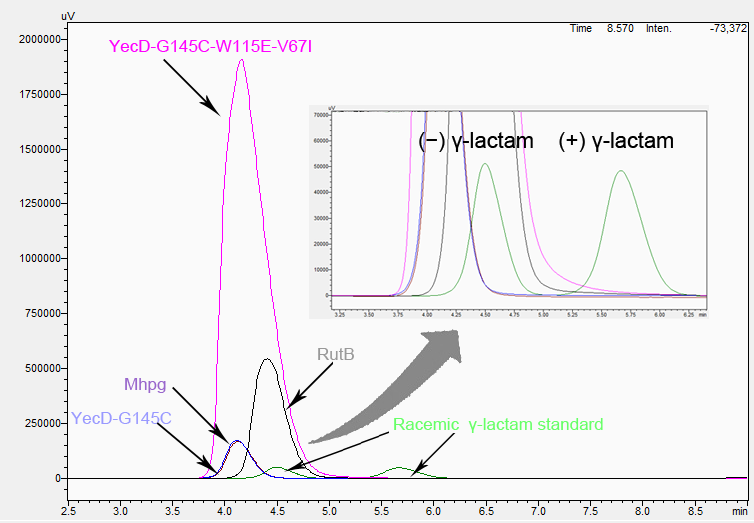


Fig. S1 Chiral HPLC analysis of scale-up transformation catalyzed by different enzymes

Muftic, M.K. 1964. A NEW PHENOL-HYPOCHLORITE REACTION FOR AMMONIA. *Nature*, **201**, 622-3.
